# Health-Related Quality of Life in Very Long-Term Cancer Survivors 14–24 Years Post-Diagnosis Compared to Population Controls: A Population-Based Study

**DOI:** 10.3390/cancers13112754

**Published:** 2021-06-01

**Authors:** Daniela Doege, Melissa S. Y. Thong, Linda Weißer, Lena Koch-Gallenkamp, Lina Jansen, Heike Bertram, Andrea Eberle, Bernd Holleczek, Alice Nennecke, Ron Pritzkuleit, Annika Waldmann, Sylke Ruth Zeissig, Hermann Brenner, Volker Arndt

**Affiliations:** 1 Unit of Cancer Survivorship, Division of Clinical Epidemiology and Aging Research, German Cancer Research Center (DKFZ), 69120 Heidelberg, Germany; m.thong@dkfz.de (M.S.Y.T.); v.arndt@dkfz.de (V.A.); 2 Division of Clinical Epidemiology and Aging Research, German Cancer Research Center (DKFZ), 69120 Heidelberg, Germany; l.weisser@dkfz.de (L.W.); l.koch-gallenkamp@dkfz.de (L.K.-G.); l.jansen@dkfz.de (L.J.); h.brenner@dkfz.de (H.B.); 3 Medical Faculty Heidelberg, University of Heidelberg, 69120 Heidelberg, Germany; 4 Cancer Registry of North Rhine-Westphalia, 44801 Bochum, Germany; Heike.Bertram@krebsregister.nrw.de; 5 Bremen Cancer Registry, Leibniz Institute for Prevention Research and Epidemiology—BIPS, 28359 Bremen, Germany; eberle@leibniz-bips.de; 6 Saarland Cancer Registry, 66024 Saarbrücken, Germany; B.Holleczek@gbe-ekr.saarland.de; 7 Hamburg Cancer Registry, 20539 Hamburg, Germany; alice.nennecke@bwfgb.hamburg.de; 8 Cancer Registry of Schleswig-Holstein, 23538 Lübeck, Germany; Ron.Pritzkuleit@uksh.de; 9 Institute for Social Medicine and Epidemiology, University of Lübeck, 23538 Lübeck, Germany; annika.waldmann@uksh.de; 10 Cancer Registry of Rhineland-Palatinate, 55116 Mainz, Germany; zeissig@krebsregister-rlp.de; 11 Division of Preventive Oncology, German Cancer Research Center (DKFZ), 69120 Heidelberg, Germany; 12 German Cancer Consortium (DKTK), German Cancer Research Center (DKFZ), 69120 Heidelberg, Germany

**Keywords:** cancer survivorship, health-related quality of life, population-based, long-term effects, age effects

## Abstract

**Simple Summary:**

Little is known about the health-related quality of life in very long-term cancer survivors 10 and more years post-diagnosis. Therefore, we compared the health-related quality of life of survivors of breast, colorectal, and prostate cancer (14–24 years post-diagnosis) with that of same-aged non-cancer controls, according to age, sex, and disease status (disease-free vs. stage IV, recurrence, metastasis, or second cancer). We found that the overall global health status/quality of life of cancer survivors more than a decade after diagnosis was slightly higher than that of population controls of the same age, but more symptoms and lower functioning were reported. Differences were small but statistically significant. Results differed by age, sex, and disease status. The findings point out the need for a comprehensive survivorship care program in order to monitor and treat potential late and long-term effects after the diagnosis and treatment of cancer. Survivorship care should be risk-adapted to survivors’ needs according to sociodemographic and clinical factors.

**Abstract:**

(1) Background: Little is known about the health-related quality of life (HRQoL) in very long-term cancer survivors (VLTCS) 10 and more years post-diagnosis. The objective was to compare cancer survivors’ HRQoL 14–24 years post-diagnosis with that of same-aged non-cancer controls, stratified by age, sex, and disease status (disease-free vs. stage IV, recurrence, metastasis, or second cancer). (2) Methods: We recruited 2704 very long-term survivors of breast, colorectal and prostate cancer, and 1765 controls in German multi-regional population-based studies. The HRQoL was assessed by the European Organization for Research and Treatment of Cancer Quality of Life Questionnaire Core 30 (EORTC QLQ-C30). Differences in the HRQoL were estimated with multiple regression, controlling for age, sex (where appropriate), and education. (3) Results: The overall global health status/quality of life of VLTCS more than a decade after diagnosis was slightly higher than that of population controls of the same age, but more symptoms and lower functioning were reported. Differences were small but statistically significant. Results differed by age, sex, and disease status. (4) Conclusions: The findings point out the need for a comprehensive survivorship care program in order to monitor and treat potential late and long-term effects after the diagnosis and treatment of cancer. Survivorship care should be risk-adapted to survivors’ needs according to sociodemographic and clinical factors.

## 1. Introduction

The number of cancer survivors is increasing due to earlier detection, better treatment options, and demographic aging [1,2]. Cancer may be considered a chronic disease [3,4] as it can affect survivors’ lives and health-related quality of life (HRQoL) for years [5,6,7].

A meta-analysis that included more than 60 studies found that, on average, long-term cancer survivors (LTCS) showed medium-to-large detriments in most HRQoL domains compared to the normative data, with the largest detriments in role-physical health (i.e., difficulties accomplishing activities of daily living and working) [3]. However, the studies included in the review were heterogeneous regarding their definitions of long-term cancer survivorship (participants were 2–26 years post-diagnosis), and many did not include population controls or compared the HRQoL of LTCS with published normative data of specific questionnaires that do not take into account the age structure of LTCS [3]. Furthermore, long-term survivorship often includes recurrence and relapse states [4], which should also be considered when analyzing the HRQoL.

The CAESAR study (Cancer Survivorship—a Multi-regional Population-Based Study) assessed the HRQoL in a cohort of breast, colorectal, and prostate cancer survivors at two points in time and compared it to cancer-free controls [8]. For the initial round, the global health status/overall QoL in cancer survivors 5–16 years post-diagnosis was comparable to population norms, but deficits in social, role, emotional, cognitive, and physical functioning, as well as specific symptoms and financial difficulties, were more prevalent in this group of cancer survivors [8].

Other studies on LTCS, i.e., those having survived 5 or more years past diagnosis [9,10,11,12,13], showed comparable results. In another German study, LTCS of mixed cancers 5 and 10 years past diagnosis reported lower functioning and a higher symptom burden in fatigue, insomnia, and pain than the general population [14]. Cancer-free LTCS reported a higher HRQoL than those LTCS who had recurrence of the primary cancer, metastasis, or a second primary cancer [14]. Regarding tumor sites, long-term breast cancer survivors, on average, reported the lowest functioning, while long-term prostate cancer survivors reported the highest functioning and the lowest symptom burden [14]. In an Italian study, LTCS 5 and more years cancer free and treatment free reported lower vitality, physical functioning, and physical and emotional role functioning compared with cancer-free population controls [15].

To improve the survivorship care, whether detriments in the HRQoL can also be found in very LTCS (VLTCS) who are 10 or more years post-diagnosis is of interest. A population-based-study on colorectal cancer survivors 15 years post-diagnosis found that VLTCS reported a significantly higher social quality of life and lower depression compared with age-matched controls, while there was no difference in fatigue and urinary functioning and only small detriments for VLTCS in bowel function compared with controls [16]. Regarding the average age of VLTCS, age-adjusted comparisons with cancer-free population controls are essential to distinguish the long-term effects of cancer from those of normal aging. However, pertinent studies that specifically addressed HRQoL and that included VLTCS often did not include age-matched controls [17,18,19,20,21,22], focused on specific cancer sites [16,18,19,20,21,22,23], or were limited to certain age ranges [18,19,20,24], UICC/cancer stages [19,22,24], or treatments [19,23]. As such, more research is needed to understand the moderators of the HRQoL in VLTCS [3].

It is important to study VLTCS in order to increase awareness and improve clinical policies and guidelines for the potential needs of this growing population. Regular follow-up care might have ended for most VLTCS, especially if disease free, and transition back to primary care in terms of a shared care model is not standardized in Germany [25]. Further, for clinicians and for VLTCS themselves, it might be helpful to identify prevalent symptoms and impairments in functioning as potential long-term/late effects of the cancer. In addition, information that certain problems might be more age related than cancer related could support VLTCS’ coping and adjustment process.

For these reasons, the objective of this study was to study cancer survivors’ HRQoL 14–24 years post-diagnosis, namely participants of the CAESAR study who consented and were still alive at follow-up. The HRQoL of these VLTCS was compared with that of same-aged non-cancer controls. Results were also stratified by tumor, sex, and recurrence status in order to detect the differential needs of certain subgroups of VLTCS.

## 2. Materials and Methods

The study compared participants from two population-based studies, namely “Cancer Survivorship—A Multi-regional Population-Based Study” (CAESAR) and “Lebensqualität in Deutschland” (LINDE)—Quality of Life in Germany.

### 2.1. CAESAR (Cancer Survivors)

**Figure 1:**
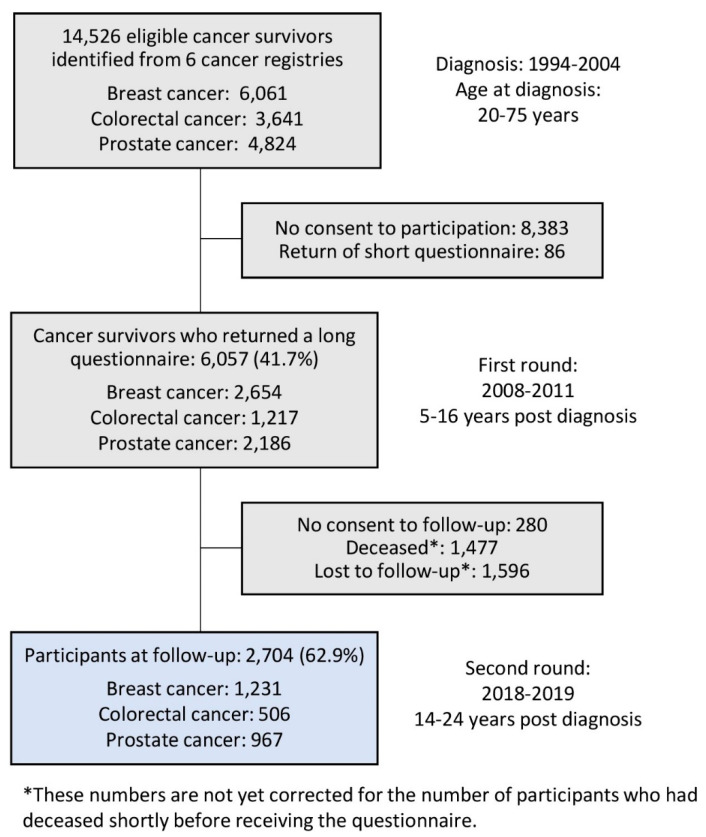
Flowchart of the CAESAR study (initial and follow-up survey).

The CAESAR study included long-term cancer survivors diagnosed between 1994 and 2004, reported to one of six participating German population-based cancer registries (Bremen, Hamburg, North Rhine–Westphalia, Rhineland–Palatinate, Saarland, and Schleswig–Holstein). Inclusion criteria were age at diagnosis of 20–75 years and a histological confirmation of breast, colorectal, or prostate cancer. Details on the first round of data collection (2008–2011, 5–16 years post-diagnosis) have been reported elsewhere [8]. The follow-up study (14–24 years post-diagnosis) was conducted between 2018 and 2019 by a postal questionnaire. Non-respondents received up to two follow-up reminder letters. Of 5777 participants with a full questionnaire of the first round who consented to be contacted again, about 4300 were still alive according to the information in the cancer registries (preliminary estimated number) and 2704 completed the full-length questionnaire at follow-up (62.9%; see Figure 1).

### 2.2. LINDE (Population Controls)

The LINDE study assessed the individual HRQoL from a representative sample of the German population. A total of 10,580 men and women, aged 18 years and above, stratified by age and sex, were randomly selected from the general German population via regional municipal offices. Data collection was conducted between 2013 and 2014. Potential participants received detailed study information and a postal questionnaire. Non-respondents received two follow-up reminder mails and a telephone contact (or one mailed reminder and a personal handover of the questionnaire, if necessary). In total, 2849 individuals participated (response rate 29%). As a control group for the current study, we included those participants with a full-length questionnaire who were cancer free and of comparable age. Participants with a self-reported history of cancer (*n* = 297) and who were younger (<40 years, *n* = 360) or older than the VLTCS sample (>99 years, *n* = 2) were excluded. Finally, 1765 LINDE participants remained and served as controls for this analysis.

### 2.3. Measurements

The HRQoL was assessed with the internationally validated European Organization for Research and Treatment of Cancer (EORTC) QLQ-C30 questionnaire. This 30-item questionnaire consists of a global health/quality-of-life (QoL) scale, five functional scales (physical, role, emotional, cognitive, social), and nine items/scales on symptoms and financial difficulties. Answers range from 1 (very poor) to 7 (excellent) for items in the global health/QoL scale and from 1 (not at all) to 4 (very much) for all other scales. Linear transformation of raw scores to a scale of 0–100 was performed according to the EORTC scoring manual [26]. High scores on the global health/QoL and functioning scales indicate better functioning. On the symptom and financial difficulty items/scales, a high score represents a greater burden.

### 2.4. Sociodemographic and Clinical Data

In both studies, the questionnaires included sociodemographic and clinical information such as marital status, education, employment, and comorbidities. Information on treatment and on recurrence, metastasis, and new primary cancers since initial diagnosis was also assessed via self-report. At time of initial survey, all cancer registries involved in the CAESAR study provided additional clinical information on cancer survivors, such as year of diagnosis and cancer stage.

### 2.5. Statistical Analyses

We compared respondents and non-respondents regarding age, sex, and clinical characteristics in order to detect response bias. Differences were determined with chi-square tests.

Sociodemographic differences between VLTCS and population controls were determined with chi-square tests. The age distribution of population controls reflected a stratified sampling scheme but was still significantly different from that of VLTCS. Therefore, we dummy-coded all characteristics and used direct standardization to compare further sample characteristics using the age and sex distribution of VLTCS as a standard. Age at the survey was categorized as follows: 40–59, 60–69, 70–79, and 80–99 years. Cochran–Mantel–Haenszel (CMH) tests were used to determine the statistical significance of differences between VLTCS and population controls, controlling for age and sex. Clinical characteristics of disease-free VLTCS and those with active disease were compared with chi-square tests.

Least square means of HRQoL scores were adjusted for age (categories of 5 years: 40–44, 45–49, … 95–99 years), sex (where appropriate), and education (≤9, 10–11, ≥12 years) using multiple linear regression. Employment status, marital status, and comorbidity also differed between cancer survivors and controls. These variables, however, were not included for adjustment, as they reflect the situation at the time of the survey and potential differences could also be a consequence of the cancer among VLTCS. VLTCS were further stratified into two subgroups by recurrence status, whereby disease-free survivors were categorized as stage I–III at diagnosis and no further report of disease progression, and the other group included survivors fulfilling at least one of the following criteria: stage IV at diagnosis or self-reporting of any recurrence, metastasis, or second cancer after the year of the study cancer.

We employed multiple imputation based on the Markov chain Monte Carlo method with 25 repetitions to reduce possible bias due to missing values (in general, less than 10%). All analyses were conducted with SAS version 9.4 for Windows (SAS Institute Inc., Cary, NC, USA). A *p*-value < 0.05 (two-sided) was considered statistically significant. The *p*-values were not adjusted for multiple testing, referring to the individual tests rather than a global test for differences.

## 3. Results

### 3.1. Non-Respondent Analysis

Compared to non-respondents, the respondents of the current follow-up round were significantly younger at diagnosis (58.7 years vs. 62.7 years, *p* < 0.05) with a slightly but significantly shorter time from diagnosis (8.0 years vs. 8.2 years, *p* < 0.05). There was no significant difference in sex. Respondents were more often diagnosed with stage I and II disease (25.7 vs. 22.2%) and less frequently with stage IV (2.3 vs. 3.8%, *p* < 0.05). Among respondents compared with non-respondents, breast cancer was slightly more frequent (45.6 vs. 42.4%) and colorectal cancer was less frequent (18.7 vs. 20.9%, *p* < 0.05), while there was no difference in prostate cancer (data not shown).

### 3.2. Study Population Characteristics

The mean age was 75.2 years for VLTCS and 63.4 years for controls, and females were slightly overrepresented in both groups. After adjustment for age and sex, VLTCS compared with controls reported statistically significant but small differences in employment, were more often married or partnered, had children less often, and reported fewer comorbidities (Table 1). Participants of both groups had predominantly German nationality (VLTCS 99%, controls 97%, data not shown). 

Among VLTCS with active disease compared to disease-free survivors, breast cancer was less frequent (39.4 vs. 47.8%) and prostate cancer was more frequent (41.1 vs. 33.8%). VLTCS with active disease were more often male. Comparing treatments, we found only small differences, i.e., those with active disease compared to disease-free survivors reported higher rates of radiotherapy (61.1 vs. 55.8%) and endocrine therapy (41.2 vs. 32.2%, Table 2). 

### 3.3. HRQoL of VLTCS and Population Controls

VLTCS reported a better global health status/QoL than controls but lower social functioning (Table 3). Regarding symptoms, VLTCS reported less pain but also more fatigue, nausea and vomiting, dyspnea, insomnia, constipation, and diarrhea. The pattern was similar when we stratified our sample of survivors by tumor type. Constipation and diarrhea were significantly higher in all subgroups when compared to controls but were highest in female colorectal cancer survivors (data not shown).

**Figure 2:**
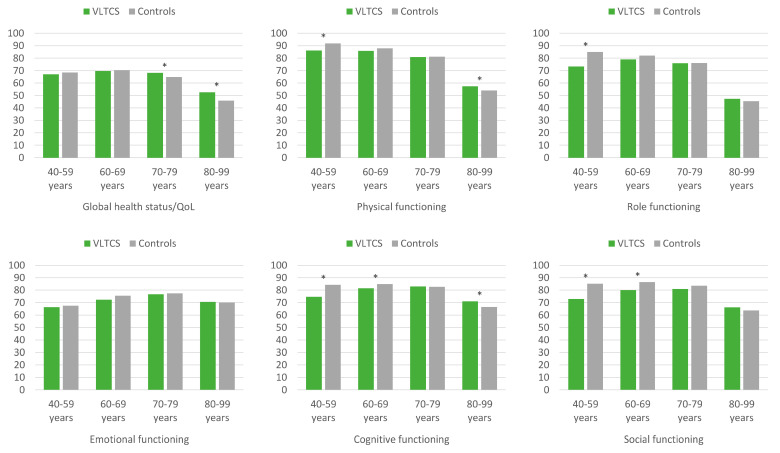
EORTC QLQ-C30 global health status/QoL and functioning scores of very long-term cancer survivors and population controls, stratified by age and adjusted for age, sex, and education. VLTCS, very long-term cancer survivors (recruited via population-based cancer registries). Asterisks (*) mark statistically significant subgroup differences (*p* < 0.05). All results are based on 25 imputations of missing values.

When stratified by age (Figure 2), we found a more diverse picture. Compared to controls, VLTCS aged 40–59 years reported lower physical and role functioning, while VLTCS aged 40–69 years reported lower cognitive and social functioning. However, VLTCS aged 70–99 years reported a better global health status/QoL, and VLTCS aged 80–99 years reported better physical and cognitive functioning than controls.

**Figure 3:**
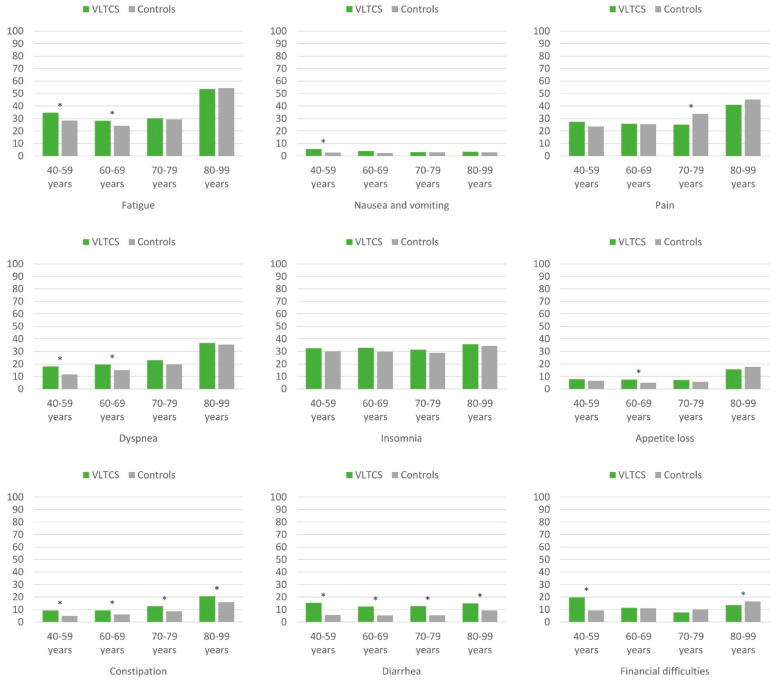
EORTC QLQ-C30 symptom items and scores of very long-term cancer survivors and population controls, stratified by age and adjusted for age, sex, and education. VLTCS, very long-term cancer survivors (recruited via population-based cancer registries). Asterisks (*) mark statistically significant subgroup differences (*p* < 0.05). All results are based on 25 imputations of missing values.

Regarding symptoms, VLTCS at age 40–69 years reported more fatigue and dyspnea compared with controls (Figure 3). Survivors aged 40–59 years reported more nausea and vomiting than controls, but in general, this symptom was rare. A lower level of pain among VLTCS compared with controls was evident in the 70–79-year age group. For constipation and diarrhea, VLTCS of all ages reported higher scores than controls, with highest differences for diarrhea at ages 40–79 years. Younger VLTCS aged 40–59 years compared with controls suffered from more financial difficulties, whereas VLTCS at age 80–99 years reported fewer financial difficulties than the control group.

### 3.4. HRQoL of Disease-Free VLTCS and Those with Active Disease

**Figure 4:**
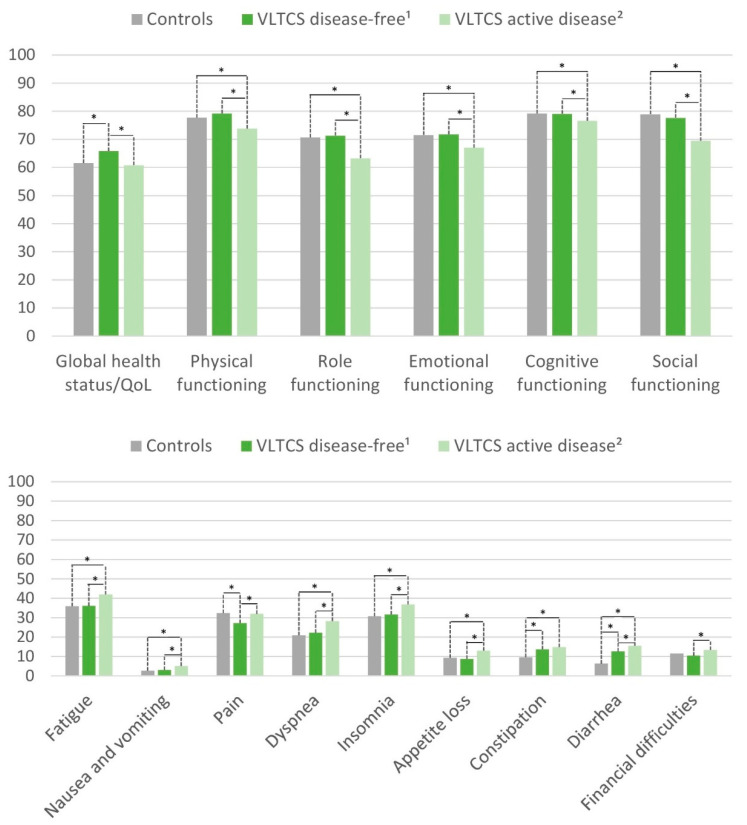
EORTC QLQ-C30 global health status/QoL, functioning scores, and symptom items and scores of long-term cancer survivors with and without active disease and of controls, adjusted for age, sex, and education. VLTCS, very long-term cancer survivors (recruited via population-based cancer registries). ^1^ Stage I–III at diagnosis and disease-free at follow-up according to self-report. ^2^ Stage IV at diagnosis or subsequent recurrence, metastasis, or second primary cancer. All differences were statistically significant (*p* < 0.05) in global comparison. Asterisks (*) mark statistically significant differences in pairwise comparison (*p* < 0.05). The spans of the lines indicate which subgroups differ significantly in pairwise comparison. For example, if the line spans three columns, it indicates a significant difference between controls and VLTCS with active disease. For example, global health status/QoL: Disease-free VLTCS report a higher QoL than controls and VLTCS with active disease, whereby controls and VLTCS with active disease are not significantly different. All results are based on 25 imputations of missing values.

We further stratified VLTCS into disease-free and active-disease groups, as shown in Figure 4. All differences were statistically significant (*p* < 0.05) in global comparison. Subsequently, we calculated contrasts to evaluate subgroup differences. The advantage of VLTCS compared with controls in the global health status/QoL was evident only for disease-free survivors, whereas survivors with active disease did not differ significantly from controls. VLTCS with active disease reported lower physical, role, emotional, cognitive, and social functioning than both other groups. Regarding symptoms, only disease-free survivors reported lower pain, while survivors with active disease did not differ significantly from controls. VLTCS with active disease reported more fatigue, nausea and vomiting, dyspnea, insomnia, appetite loss, and financial difficulties than disease-free survivors and controls. For these scales, disease-free survivors did not differ significantly from controls. In contrast, constipation and diarrhea were reported more frequently not only by VLTCS with active disease compared with controls but also by disease-free VLTCS compared with controls.

### 3.5. HRQoL of Female and Male VLTCS

**Figure 5:**
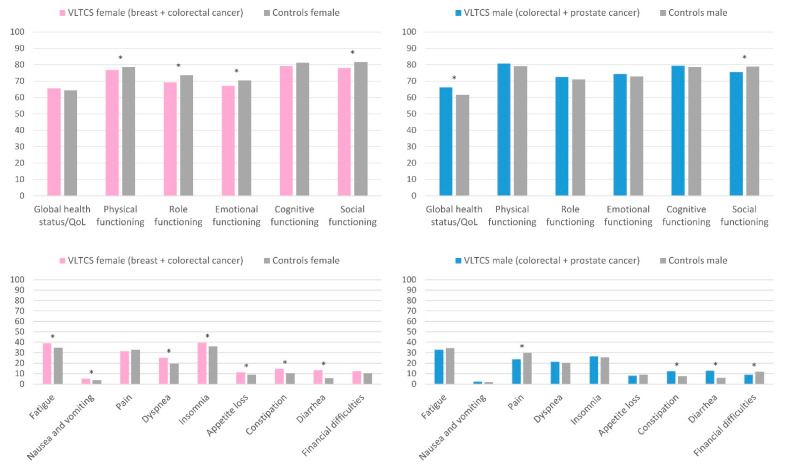
EORTC QLQ-C30 global health status/QoL, functioning scores, and symptom items and scores of female long-term cancer survivors compared with female controls and of male long-term cancer survivors compared with male controls, adjusted for age and education. VLTCS, very long-term cancer survivors (recruited via population-based cancer registries). Asterisks (*) mark statistically significant subgroup differences (*p* < 0.05).

We further stratified our samples of VLTCS and controls into female and male groups to compare the HRQoL in these subgroups separately (Figure 5). Female VLTCS (breast and colorectal cancer) compared with female controls reported lower physical, role, emotional, and social functioning (all statistically significant but small). They further reported more fatigue, nausea and vomiting, insomnia, appetite loss, constipation, dyspnea, and diarrhea.

Male VLTCS (colorectal and prostate cancer) compared with male controls reported a better global health status/QoL and less pain and fewer financial difficulties. However, they also reported lower social functioning and more constipation and diarrhea.

## 4. Discussion

Our study analyzed the HRQoL of VLTCS more than a decade after diagnosis, as there is a lack of research in this area and as it is essential for clinicians to learn about the needs of this growing and understudied group to improve clinical guidelines and develop care policies. We analyzed the functioning and symptoms of VLTCS in relation to cancer-free population controls in order to distinguish the long-term/late effects of the cancer from the effects of aging. On average, VLTCS rated their global health status/QoL slightly better than age- and sex-adjusted controls. Looking closer at subgroups of VLTCS, these better ratings compared to controls were only reported by survivors aged >70 years, by those without active disease, and/or by male survivors. The other subgroups of VLTCS had a global health status/QoL comparable to controls. The finding that VLTCS reported an overall good global health status/QoL despite specific detriments is in line with previous studies on LTCS [8,10]. Male VLTCS in the current study also reported less pain, which has been previously found for long-term prostate cancer survivors [27,28] and might be explained by a response shift [28], meaning that cancer survivors compare their current level of pain to the level of pain they experienced through cancer and its treatment and thus rate it different compared to the time before cancer diagnosis and cancer-related treatment. 

The fact that the global health status/QoL of VLTCS was comparable or even better than that of controls is a sign that in general, most VLTCS might have successfully adapted to their cancer. 

Regarding functioning, VLTCS reported lower social functioning than controls, which can be attributed to survivors with active disease status. Cancer recurrence is associated with distress [29], and its treatment might have side effects that potentially affect partnership, sexuality, and social contact (e.g., diarrhea). 

Regarding symptoms, diarrhea and constipation were the symptoms that were more frequent in all subgroups compared with controls, irrespective of sex, age, and tumor site, and including disease-free survivors. Dietary counseling and medication might be recommended according to clinical guidelines for long-term colorectal cancer survivors [30].

Some symptoms and functioning detriments were only reported by certain subgroups; for instance, financial difficulties were mainly reported by the youngest survivor group (<59 years). These survivors are still of working age and might have encountered problems returning to their previous work or returning to work at all [31]. Survivors with active disease also reported more financial problems than disease-free survivors. This is in line with the literature that financial toxicity includes more than care costs and remains an important topic for VLTCS, implying that monitoring of socioeconomic problems is warranted [32].

Female cancer survivors and male cancer survivors reported different HRQoL detriments compared with female and male controls, respectively. It has been suggested before that age and sex should be considered when interpreting HRQoL scores in cancer patients or survivors [15,33]. This study is the first one to report gender differences in VLTCS in a large sample, compared to controls and adjusted for age. Detriments in physical, role, and emotional functioning, as well as fatigue, insomnia, dyspnea, and appetite loss, were significantly higher in female VLTCS compared with female controls. Male VLTCS reported more financial difficulties than controls, which might be due to traditional role models with men being the main earners in the household. As the effect of sex and tumor site cannot be disentangled in breast and prostate cancer survivors, we analyzed the subgroup of only colorectal cancer survivors and found comparable patterns regarding female and male survivors compared with controls. Nevertheless, further studies should explore the HRQoL in other cancer types. 

VLTCS and controls also differed in comorbidities in our study, and we did not adjust for comorbidity, as our data was cross-sectional and certain comorbidities might reflect long-term adverse effects of cancer treatment. Nevertheless, comorbidities might be a problem, especially for VLTCS, as it has been shown that cancer survivors 10–14 years post-diagnosis are at higher risk of developing comorbidities compared with those only 4–9 years post-cancer diagnosis, after adjusting for age [34]. A higher comorbidity burden might also affect the HRQoL.

The limitations of the study include the possibility of healthy survivor bias. The average age of VLTCS at the time of follow-up was 75 years, and the response rate (preliminary, due to delays in reporting of vital status) was 63%. Cancer survivors of a higher age, with a longer time period since diagnosis, or with poorer health are generally less likely to participate in cancer survivorship studies [8]. Non-response might have resulted in an overestimation of the observed HRQoL in participating VLTCS. Likewise, non-participation among non-cancer controls, where the response rate was 29%, might have also introduced bias [34]. However, as comorbidities were less frequent among VLTCS, it is likely that healthy survivor bias is more relevant in VLTCS, which, in turn, corresponds to a potential underestimation of the true difference between survivors and controls. The results were based on cross-sectional analysis rather than on longitudinal development of individuals. Thus, the relationships between age, active disease status, sex, and the HRQoL cannot be interpreted causally. Higher survival rates among lower cancer stages could have biased the comparison between disease-free VLTCS and those with active disease. Furthermore, we had missing data on relevant variables such as cancer stage because this variable was not fully reported to the cancer registries in the period when cancer was diagnosed. We imputed missing data and ran sensitivity analyses, which showed that results derived from multiple imputations were similar to those from non-imputed data. For VLTCS with active disease, data on recurrence, metastasis, and second cancers were self-reported and might underlie recall bias. In addition, we did not control for the date of recurrence, metastasis, or a second cancer, as the exact year was often not reported by participants. Further, we summarized participants who had any sign of active disease into one potentially heterogeneous subgroup. Nevertheless, we think it is important to include these persons as cancer survivors in the overall analyses. Further studies might analyze VLTCS according to stage, recurrence, metastasis, or a second cancer in more detail.

The strengths of this study include the population-based recruitment of both the VLTCS and the non-cancer control group with comparable data collection mode, which resulted in diverse cohorts with respect to sociodemographics, treatments, and stages. The large sample size allowed for age stratification, leading to a more diverse picture of HRQoL aspects in the analyzed subgroups.

Most differences between VLTCS and controls found in this study were small and were observed only in certain age groups. Further studies might analyze whether the small but significant detriments in HRQoL domains, especially for survivors with active disease, are associated with further outcomes like mortality.

## 5. Conclusions

VLTCS and health-care providers should be aware of possible late effects and chronic long-term sequelae in vulnerable subgroups of cancer survivors. Potential long-term and late effects in cancer survivors should be monitored and treated in comprehensive survivorship care before they become chronic. This survivorship care, which should also exceed the current practice of follow-up limited to the first 5 years post-diagnosis, should be tailored to the needs of clinical and sociodemographic subgroups of cancer survivors, considering age, sex, and recurrence status. While, e.g., survivors younger than 60 years and male survivors might benefit from return to work or from financial support in order to improve their role and social functioning, survivors older than 80 years might benefit from interventions that support their physical and cognitive functioning. Female survivors might benefit from interventions to improve sleep and to reduce fatigue.

In conclusion, it is heartening that most VLTCS report a comparable overall health status/QoL to non-cancer controls. Nevertheless, persistent symptoms and functioning deficits highlight the importance to identify and assist vulnerable survivors to cope better in survivorship.

## Figures and Tables

**Table 1:** Characteristics of very long-term cancer survivors and population controls.

Characteristics	Cancer Survivors		Population Controls			p_crude_ (χ^2^)	p_adj_ (CMH)
	*n*	%	*n*	%	%_adj_^a^		
**Total**	2704	100.0	1765	100.0			
**Age (at survey)**							
40–59 years	169	6.3	726	41.1	6.3	**<0.0001**	-
60–69 years	468	17.3	399	22.6	17.3		
70–79 years	1114	41.2	345	19.5	41.2		
80–99 years	953	35.2	295	16.7	35.2		
**Mean age (SD)**	75.2	(8.7)	63.4	(13.8)			
**Sex**							
Female	1438	53.2	942	53.4	53.2	0.90	**-**
Male	1266	46.8	823	46.6	46.8		
**Education**							
≤9 years	1267	46.9	671	38.0	49.5	**<0.0001**	0.08
10 years	707	26.1	484	27.4	22.0		
≥12 years	730	27.0	610	34.6	28.5		
**Employment (at survey)**							
Full-time	90	3.3	455	25.8	5.2	**<0.0001**	**<0.0001**
Part-time	139	5.1	255	14.4	5.6		
Unemployed	6	0.2	53	3.0	1.3		
Housewife	286	10.6	180	10.2	13.3		
(Early) Retirement	1978	73.1	752	42.6	70.9		
Other	47	1.7	50	2.8	2.3		
Multiple answers	158	5.8	20	1.1	1.4		
**Having a partner (at survey)**	2046	75.7	1335	75.7	69.8	0.96	**0.0002**
**Having children**	2317	85.7	1503	85.2	89.8	0.61	**0.003**
**Current marital status**							
Unmarried	118	4.4	166	9.4	3.9	**<0.0001**	**<0.0001**
Married	1914	70.8	1180	66.8	63.8		
Divorced	183	6.8	167	9.5	6.6		
Widowed	489	18.1	252	14.3	25.7		
**History of comorbidities (self-report)**							
Coronary heart disease	254	9.4	157	8.9	13.9	0.63	**<0.0001**
Osteoporosis	365	13.5	167	9.5	15.7	**<0.0001**	0.33
Diabetes	378	14.0	222	13.0	17.7	0.19	**0.01**
Chronic back pain	914	33.8	567	32.1	38.6	0.41	**0.02**
Depression (ever)	304	11.3	285	16.1	14.1	**<0.0001**	**0.04**

^a^ Rates of population controls standardized by age and sex distribution of cancer survivors cohort. χ^2^ = chi-square tests. CMH, Cochran–Mantel–Haenszel statistics. All results are based on 25 imputations of missing values. Numbers might not add up to the total sample size due to rounding of multiple imputation results. Percentages might not add up to 100% due to rounding of percentages. **Bold** *p*-values show statistically significant differences (*p* < 0.05).

**Table 2:** Characteristics of very long-term cancer survivors and disease-free survivors versus survivors with stage IV at diagnosis or with subsequent recurrence, metastasis, or second cancer (active disease).

Characteristics	Disease-Free Survivors		Survivors with Active Disease		
	*n*	%	*n*	%	*p* (χ^2^)
**Total**	1972	72.9	732	27.1	
**Age at (current) survey**					
40–59 years	118	6.0	51	7.0	0.10
60–69 years	362	18.4	106	14.5	
70–79 years	807	40.9	307	41.9	
80–99 years	685	34.7	268	36.6	
**Mean age (SD)**	75.0	(8.6)	75.7	(8.7)	
**Tumor**					
Breast cancer	942	47.8	289	39.4	**0.0003**
Colorectal cancer	363	18.4	143	19.5	
Prostate cancer	666	33.8	301	41.1	
**Sex**					
Female	1098	55.7	340	46.5	**<0.0001**
Male	874	44.3	392	53.5	
**Stage at diagnosis**					
I	611	31.0	182	24.9	**<0.0001**
II	973	49.3	317	43.2	
III	389	19.7	159	21.7	
IV			75	10.2	
**Recurrence/metastasis after diagnosis**			455	62.2	
**Second primary tumor**			317	43.3	
**Primary therapy**					
Surgery	1538	78.0	561	76.7	0.47
Radiotherapy	1100	55.8	447	61.1	**0.01**
Chemotherapy	806	40.9	273	37.3	0.09
Hormone (endocrine) therapy	595	32.2	263	38.5	**0.003**
Immuno-/Antibody therapy	109	6.3	41	6.5	0.87

χ^2^ = chi-square tests. All results are based on 25 imputations of missing values. Numbers might not add up to the total sample size due to rounding of multiple imputation results. Percentages might not add up to 100% due to rounding of percentages. **Bold** *p*-values show statistically significant differences (*p* < 0.05).

**Table 3:** Health-related quality of life of very long-term cancer survivors and population controls, adjusted for age, sex, and education.

EORTC QLQ-C30 Scales/Scores	Cancer Survivors		Population Controls			
	Mean	SE	Mean	SE	Diff.	*p* (glm)
Global health status/QoL	64.4	1.2	61.5	1.2	2.9	**0.0002**
Physical functioning	77.7	1.1	77.6	1.1	0.0	0.97
Role functioning	69.0	1.5	70.6	1.6	−1.5	0.13
Emotional functioning	70.4	1.3	71.5	1.3	−1.1	0.20
Cognitive functioning	78.4	1.2	79.2	1.2	−0.8	0.30
Social functioning	75.3	1.5	78.8	1.5	−3.5	**0.0003**
Fatigue	37.8	1.4	36.0	1.4	1.8	**0.045**
Nausea and vomiting	3.6	0.6	2.7	0.6	0.9	**0.02**
Pain	28.6	1.6	32.5	1.6	−3.9	**0.0003**
Dyspnea	24.0	1.5	21.0	1.6	3.0	**0.003**
Insomnia	33.2	1.7	30.8	1.8	2.4	**0.04**
Appetite loss	10.0	1.1	9.4	1.1	0.6	0.40
Constipation	14.0	1.3	9.6	1.3	4.4	**<0.0001**
Diarrhea	13.5	1.1	6.4	1.1	7.1	**<0.0001**
Financial difficulties	11.3	1.2	11.5	1.2	−0.3	0.73

EORTC QLQ-C30, European Organization for Research and Treatment of Cancer Quality of Life Questionnaire Core 30; SE, standard error; glm, generalized linear model. All results are based on 25 imputations of missing values. **Bold** *p*-values show statistically significant differences (*p* < 0.05).

## Data Availability

The data presented in this study are available on request from the corresponding author.
